# Left Ventricle Non-Compaction Cardiomyopathy Admitted With Multiorgan Failure: A Case Report

**DOI:** 10.7759/cureus.8787

**Published:** 2020-06-23

**Authors:** Luis F Álvarez Pérez, Jorge E Sandelis Pérez, Juan Nieves-Rivera, Hilton Franqui

**Affiliations:** 1 Internal Medicine, University of Puerto Rico, Medical Sciences Campus, San Juan, PRI; 2 Cardiology, University of Puerto Rico, Medical Sciences Campus, San Juan, PRI

**Keywords:** congenital, cardiomyopathy, left ventricle, non-compaction, heart failure, ventricular tachycardia

## Abstract

Left ventricle non-compaction (LVNC) is a rare congenital cardiomyopathy characterized by thickened myocardium due to an arrest of the normal compaction of the embryonic sponge-like meshwork of myocardial fibers.

We present a 40-year-old man with no known systemic illnesses admitted with cardiogenic shock and multiorgan failure. Echocardiogram revealed severe enlargement of all four chambers with left ventricular ejection fraction (LVEF) <10%. Cardiac magnetic resonance imaging (CMR) showed hypertrabecular left ventricular myocardium with a ratio of non-compact to compact myocardium of 2.3, diffuse myocardial thinning, and a 16-mm left ventricular thrombus. These findings were compatible with LVNC. The patient was treated with intravenous inotropic vasopressors for cardiogenic shock and enoxaparin as bridging for warfarin to a goal of INR 2.0-3.0. Due to refractory heart failure (HF) and dependency on inotropic support, the patient was placed on the waiting list for a heart transplant. Unfortunately, 27 days after admission, he presented ventricular tachycardia arrest and did not respond to aggressive advanced cardiac life support measures.

A high index of suspicion is required for the early diagnosis, which in turn allows the physician to prevent complications of this condition. There is no specific therapy, so management is directed toward the clinical manifestations including HF, arrhythmias, and systemic embolic events. Heart transplantation is the only definitive treatment.

## Introduction

Left ventricle non-compaction (LVNC) is a very rare congenital cardiomyopathy characterized by thickened myocardium due to an arrest of the normal compaction of the embryonic sponge-like meshwork of myocardial fibers [1-4]. The first isolated case was described in 1984 by Engberding, and its prevalence among patients undergoing echocardiography is estimated at 0.014-1.3% [5-7] and 3-4% in patients with heart failure (HF) [8].

A high index of suspicion is required for the early diagnosis, which in turn allows the physician to prevent complications of this condition such as fatal arrhythmias, thromboembolic phenomenon, severe HF, and sudden cardiac death.

We present a complex case admitted with cardiogenic shock in the setting of de novo HF secondary to LVNC.

## Case presentation

**Figure 1:**
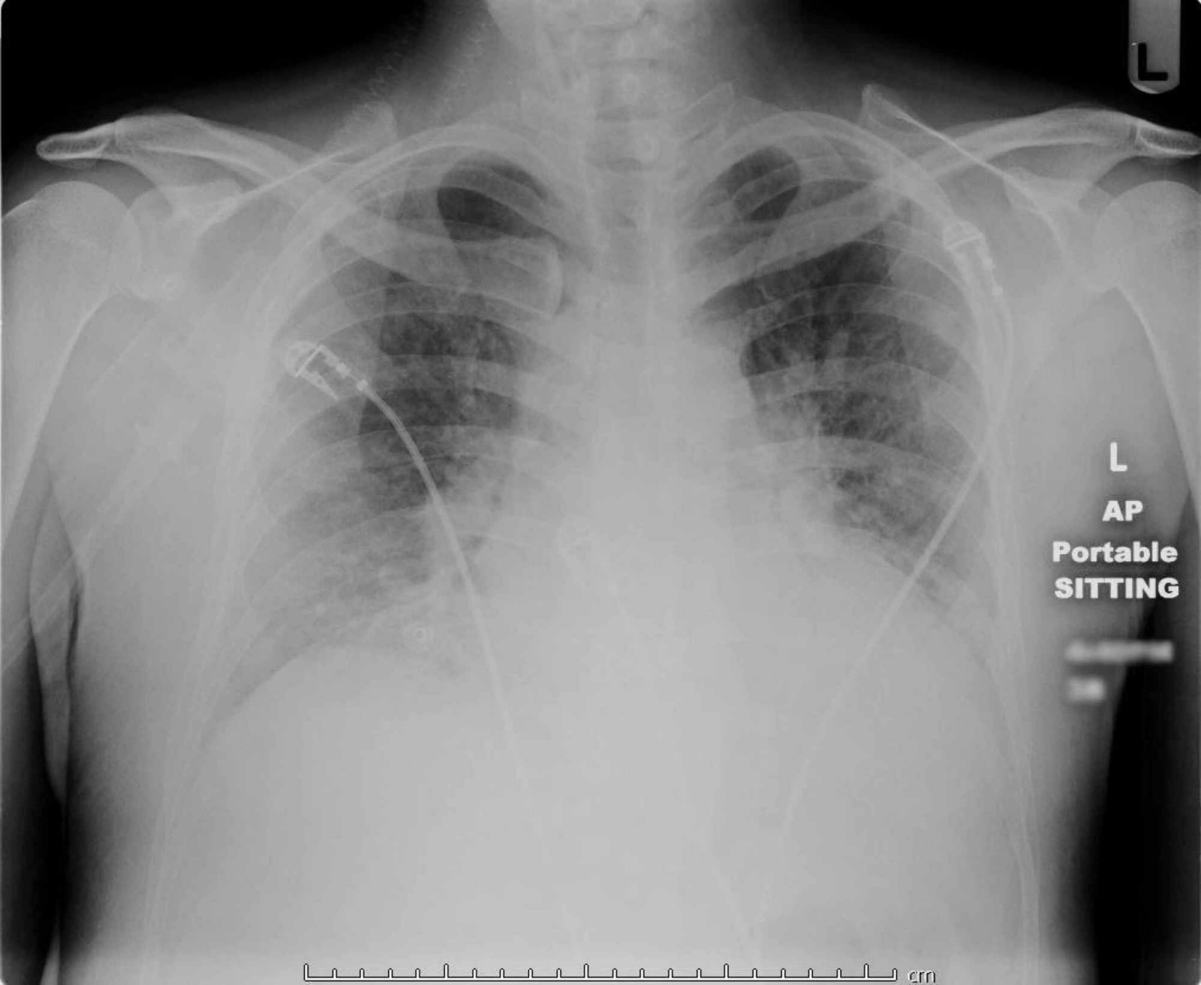
Portable CXR (single view) The X-ray shows cardiomegaly and mild pulmonary vascular congestion centrally, indicating early CHF changes; additionally, there are no consolidations, no pneumothorax, and the anterior pleura spaces are clear. The bones are grossly intact. CXR: chest X-ray; CHF: congestive heart failure

A 40-year-old man with no known systemic illnesses presented with a four-week history of progressive shortness of breath and lower extremity swelling. Associated symptoms included dyspnea on minimal exertion, orthopnea, and paroxysmal nocturnal dyspnea. Vital signs were remarkable for a blood pressure of 90/40 mmHg, tachycardia of 110 bpm, and pulse oximetry of 92%. Physical examination was relevant for anasarca, venous jugular pressure of 12 mmHg, diffuse bilateral inspiratory wet pulmonary crackles, a third heart sound (S3), a grade II/VI systolic murmur at the apex, and cold extremities. Laboratory tests were consistent with acute kidney injury (serum creatinine of 1.7 mg/dL and urea nitrogen of 49 mg/dl) and markedly elevated liver enzymes with aspartate aminotransferase of 5366 IU/L and alanine aminotransferase of 4967 IU/L. These findings were consistent with multiorgan failure. A chest X-ray revealed pulmonary vascular congestion (Figure 1).

**Figure 2:**
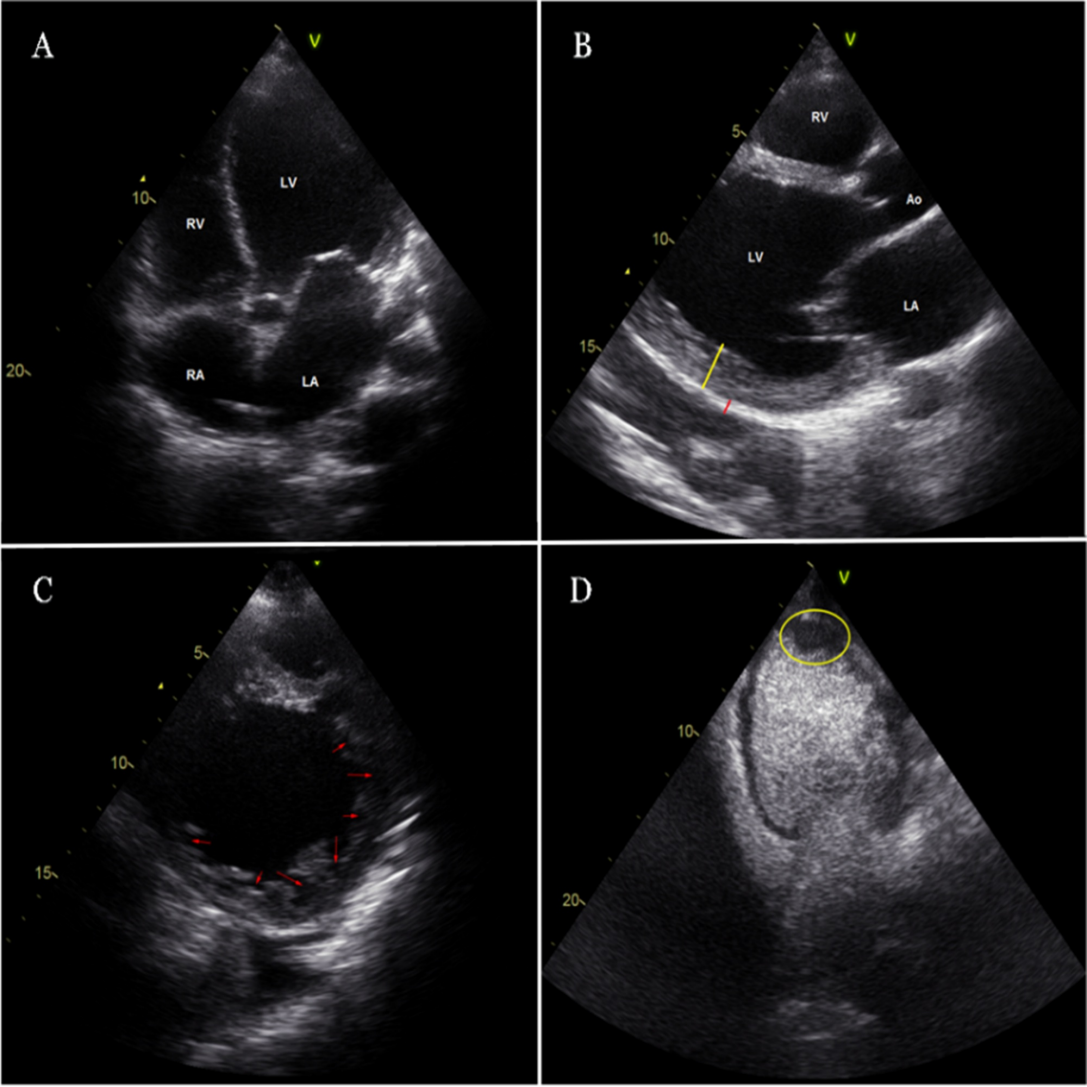
2D echocardiogram Apical four-chamber view (A) showing severe enlargement of all four chambers, a thin compacted epicardial layer (red line) and a markedly thickened endocardial layer (yellow line) with a maximum ratio of non-compacted to compacted myocardium >2:1 can be appreciated on the parasternal long-axis view (B); the parasternal short-axis view (C) is pertinent for numerous prominent trabeculations and deep recesses (red arrows). On apical two-chamber view (D) there is an apical left ventricular thrombus (yellow circle).

The echocardiogram showed severe mitral regurgitation and tricuspid regurgitation, with severe enlargement of all four chambers, and left ventricular ejection fraction (LVEF) <10% (Figure 2). The patient was admitted to the cardiac care unit.

Cardiac catheterization demonstrated normal coronary arteries and reversible mild pulmonary hypertension.

**Figure 3:**
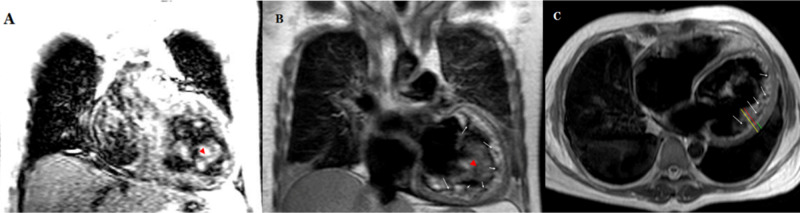
Cardiac magnetic resonance imaging (A) and (B) (coronal Views) showed left ventricular intracavitary thrombus identified in the apical lateral wall, measuring 16 mm (red arrowhead), with hypertrabeculation and deep myocardial intratrabecular recesses; (B) and (C) (white arrows), the ratio of the compacted to non-compacted myocardium was 2.3 (C) (Axial view) (lines yellow, red, and green)

Cardiac magnetic resonance imaging (CMR) (Figure 3) showed depressed biventricular systolic function (LVEF < 10%; RVEF < 15%), hypertrabecular left ventricular myocardium with a ratio of non-compact to compact myocardium of 2.3, diffuse myocardial thinning, and a 16-mm left ventricular thrombus. These findings were compatible with LVNC. As mentioned above, the patient had no history of heart disease, and no precipitating factor for his new-onset HF was identified during admission process.

Extremity venous Doppler revealed bilateral lower extremity posterior tibial and peroneal deep venous thrombosis, as well as upper extremity cephalic and basilic vein thrombosis.

The patient was treated with intravenous inotropic vasopressors for cardiogenic shock and enoxaparin as bridging for warfarin to a goal of INR 2.0-3.0. The patient was placed on the waiting list for heart transplant due to refractory HF and dependency on inotropic support. Unfortunately, 27 days after admission, he presented with ventricular tachycardia arrest and did not respond to aggressive advanced cardiac life support measures.

## Discussion

LVNC is thought to be caused by an arrest of the normal compaction of the embryonic sponge-like meshwork of interwoven myocardial fibers during embryogenic life, resulting in thickened myocardium with two layers consisting of non-compacted myocardium and a thin compacted layer of myocardium [9]. The presence of deep endomyocardial intertrabecular recesses, which are filled with blood from the ventricular cavity, is typical of this condition and predisposes to embolic events [1-4].

This condition may sporadically develop in individuals without family history, although an autosomal dominant is the most commonly observed pattern of inheritance [10]. More than 10 genetic mutations have been identified, especially in genes encoding for mitochondrial, Z-line, sarcomeric, and cytoskeletal proteins [11].

Its clinical manifestations are highly variable, ranging from no symptoms to disabling congestive HF, life-threatening ventricular arrhythmias, and systemic thromboembolism [5,12]. However, there is no gold standard for diagnosis of LVNC; the diagnosis should be suspected based on morphologic criteria for non-compaction or hypertrabeculation in patients with or without cardiac symptoms [13].

Echocardiogram is the most used imaging modality both to establish the diagnosis and for the follow-up [5,14,15]. The most widely accepted echocardiographic criteria are the Jenni Criteria (Table.1) and all four are required for diagnosis [15].

**Table 1:** Echocardiographic criteria for diagnosis of LVNC Adapted from Jenni et al. [15]. LV: left ventricular; LVNC: left ventricle non-compaction.

Jenni Criteria for LVNC
A thickened LV wall consisting of two layers: a thin compacted epicardial layer and a markedly thickened endocardial layer with numerous prominent trabeculations and deep recesses with a maximum ratio of non-compacted to compacted myocardium >2:1 at end-systole in the parasternal short-axis view.
Color Doppler evidence of flow within the deep intertrabecular recesses.
Prominent trabecular meshwork in the LV apex or midventricular segments of the inferior and lateral wall.
Compacted wall thickness ≤8.1 mm. The criterion of maximal systolic compacta thickness of ≤8.1 mm was found to be very specific for myocardial thickening in LVNC compared to normal controls or patients with aortic stenosis.

When echocardiographic findings are not definitive, CMR criteria can be used to support the diagnosis of LVNC; among them, the maximum end-diastolic non-compacted to compacted myocardial thickness ratio of >2.3 and a trabeculated LV mass >20% of global LV mass [16,17]. However, the sensitivity and specificity are complex to determine, and due to the risk of overestimation, these criteria should be used with caution.

There is no specific therapy, so management is directed toward the clinical manifestations including HF, arrhythmias, and systemic embolic events. Heart transplantation is the only definitive treatment. Attending to the risk of cardiac sudden death, implantable cardioverter-defibrillator may have a role in these cases [18].

## Conclusions

Because of its low prevalence, the diagnosis of LVNC cardiomyopathy is often missed or delayed in adults. A high index of suspicion is required for the early diagnosis, and prompt referral to the cardiologist in order to ensure the most appropriate management and follow-up of these patients.
